# Cost of comprehensive patient assistance program in early breast cancer patients

**DOI:** 10.1186/2193-1801-2-173

**Published:** 2013-04-19

**Authors:** Anne M Stey, Kezhen Fei, Rebeca Franco, Ali Mendelson, Nina A Bickell

**Affiliations:** Department of Health Policy, Mount Sinai Medical Center, New York, NY 10029 USA; 10940 Wilshire Blvd., Suite 710, Los Angeles, CA 90024 USA

## Abstract

Existing comprehensive patient assistance programs can help cancer patients overcome needs and barriers to care: yet the costs of such programs and who utilizes them is not well described. 333 women with primary early stage breast cancer in New York City either chose to attend or abstain from attending one of these programs. We obtained the operating costs of the most utilized patient assistance program. Patients who utilized patient assistance programs were significantly more likely to be English speaking (p=0.04) and have 1.5 years more advanced schooling than non-utilizers (p=0.008). At the cost of 73 dollars per hour encounters, patients reported benefiting the most from information resources (71% of patients) followed by emotional support (52% of patients); 5% of patients reported getting help with insurance, 5% with financial assistance, 4% with help obtaining medication, 2% with transportation. Existing comprehensive patient assistance programs offer informational, psychological and financial support at low cost. Patients who currently use these programs are English speakers with more education.

**Electronic supplementary material:**

The online version of this article (doi:10.1186/2193-1801-2-173) contains supplementary material, which is available to authorized users.

## Introduction

Disparities in breast cancer survival due to social determinants of health (Weir et al. 2003; Bach et al. 2002; Steinberg 2008; Bickell et al. 2006; Chen et al. 2008 Apr 2; Chu et al. 2003; Ward et al. 2008) may be reduced through timely screening and appropriate treatment. Patient outreach and navigation programs have been shown to improve rates of early detection and follow through to biopsy and treatment (Bickell 2002; Ell et al. 2007; Gabram et al. 2008; Freeman 2006; Battaglia et al. 2007). Such programs are but one type of patient assistance albeit the most researched, and often implemented by academic center clinical trials or through grant funding. Types of patient assitance tested include clinical trials of physical therapy, nurse-led follow-up, group therapy, psycho-social and educational counseling which improve women’s quality of life, presumably by improving perceived physical symptoms and psychological functioning (Cruickshank et al. 2008; Gordon et al. 2005; Meneses et al. 2007; Lemieux et al. 2006; Jacobsen et al. 2002; Mandelblatt et al. 2008; Ferrante et al. 2008; David et al. 2007). These programs tend to be heterogeneous, varying from those that provide only print materials or peer-support groups to provision of comprehensive care including professional therapists, social work, medical services, educational programs, transportation and even financial support. Programs such as patient navigation, nurse-led and outreach programs, although successful, are costly which is often unsustainable once research is over. Implementing such programs on a large scale requires an investment in infrastructure from an already constrained health system.

The strengths of using existing comprehensive patient assistance programs rather than relying on hospital-based navigiation include the following: 1) many programs use volunteer-wo/man power of breast cancer survivors who wish to “give back and help” current patients; 2) they are already present and integrated into the community, thus do not require the additional investment of hospital-based patient navigator or nurse-led intervention programs; 3) they obviate the need for hospitals to create separate salary lines to support these ancillary services to supplement a health system’s strained ability to meet breast cancer patients’ needs. Although many community patient assistance programs are available, they are often underutilized, an impetus for the parent randomized control trial from which these data derive (21). We outline the basic cost structure using a representative comprehensive volunteer led patient assistance program that was most used by the patients participating in an RCT testing the effectivness of patient assistance. We describe patients’ needs and who used patient assistance programs prior to the intervention to randomization.

## Methods

### Study protocol

We defined comprehensive patient assistance programs as programs having professional staffing and volunteer staffing with a not-for-profit status that offer a wide range of services including: outreach screening missions, educational lectures and print material, psychological counseling, peer-support groups and financial aid. The RCT from which data was obtained in this trial recruited English or Spanish speaking women who were treated surgically between October 2006 and July 2010 at eight participating New York City hospitals for new primary early stage breast cancer requiring adjuvant therapy as per national guidelines. Hospitals included four tertiary referral centers and four municipal hospitals. The trial received Institutional Review Board approval at all participating sites; informed consent was obtained from all enrollees.

### Survey instrument

Both baseline and six-month surveys assessed demographic information, health status, use of patient assistance programs as well as the number and type of needs, e.g., informational, psychosocial or practical needs. All surveys were translated to Spanish and validated through back-translation to English to ensure translation accuracy. 333 women were enrolled in the study and completed both baseline and six-month follow-up survey. Based on their needs assessment, women in the trial’s intervention arm were referred to existing comprehensive patient assistance programs; those in the control arm received an informational booklet about breast cancer treatment. This analysis reports the cost of attending existing comprehensive patient assistance program and describes who at attended at baseline prior to randomization; it does not assess the efficacy of the randomized trial. We included all women who had attended a patient assistance program from the time of diagnosis to any point prior to enrolling in the trial and categorized them in the patient assistance utilizer group. If they did not use from patient assistance program prior to enrolling, they were categorized in our non-utilizer group. Following baseline survey, participation in the programs was encouraged but not enforced. The two groups of patients who were either attendees or non-utilizers and cross tabulations and one-way ANOVAs were performed with SPSS 20.0.

### Cost and statistical analysis

Need analysis was conducted in an attempt to quantify the degree of perceived lack of social, practical or economic support. Women were separated into 0, 1, 2, and 3 needs groups depending on how they answered an initial needs survey which gauged their psychosocial, practical and financial needs (Additional file 1).

Costs were taken from the program costs’ perspective and gathered directly from a Breast Health Resource Program, an existing comprehensive patient assistance program in New York City, most frequently used by our study population (Table 1). The cost per hour assumed that each encounter utilized the full range of available services. The total annual expenditure of the program for 2008 was then divided by this number of encounters (6463). All prices were expressed in 2012 dollar and adjusted for Consumer Price Index. As per patient report, each encounter at the program lasted about an hour.

**Table 1:** **Cost per patient encounter derived from the breast health resource center 2007 fiscal year and adjusted for july 2012 inflation**

Service	Description	Cost/encounter
Patient education program lectures		
Mailing/print		$17.54
Educational materials		$3.95
Food & beverage		$10.97
Auditorium registration		$4.39
AV Rental		$1.32
Faculty/program Dev		
Personnel		
Salaries w/ Fringe: 4 CSW, 1 admin assist		$54.88
Patient assistance fund		
Clinical service (mammo,sono, clinic fee)		$0.98
Surgical biopsies/radiologic biopsies		$0.65
Post-Op Bras/prosthesis		$0.33
Wigs		$0.08
Patient transportation		$0.13
Emergency stipend (rent, utilities, food, childcare, medical insurance payments)		$0.16
Hospice/comprehensive care		$0.12
Facility costs		
Janitorial service		$1.01
Electricity		$0.28
Storage		$0.06
TV/Cable		$0.05
Telephone/DSL		$0.77
Telephone/computer connection		$1.55
Building space rental		$6.50
Administrative and operating costs		
Books (medical)		$0.04
Catering		$0.16
Conference room		$0.23
Consultants		$0.31
Print shop		$0.77
Equipment rental		$0.33
Messenger service		$0.04
Office supplies		$0.56
Photocopy mach supplies		$0.05
Postage		$0.23
Books/periodicals		$0.23
Marketing/website/promo items		$1.16
Stationery/brochures		$0.39
Subway, taxi, car		$0.39
Engineering/sign shop		$0.16
Course or conference registration		$0.19
Minor office equip/software		$0.08
Temp help		$0.08
Total annual cost from third party payer perspective		$72.90
Utilization in hours by each needs group and total cost		
0 Need	8 Hours (SD 5.4 , 95% CI 0-16)	$583.20
1 Need	27 Hours (SD 22.0, 95% CI 0-101)	$1968.30
2 Needs	21 Hours (SD 2.7, 95% CI 0-9)	$1530.90
3 Needs	19 Hours (SD 18.3, 95% CI 0-100)	$1385.10

Demonstrates the itemized costs of the most frequently accessed volunteer staffed community based comprehensive patient assistance program based on annual budget and total number of visits.

## Results

### Study population

Our study population included 21% African Americans and 31% Hispanic women. The majority of women spoke English, but 24% of women spoke primarily Spanish with 36% of women from Latin America or the Caribbean (Table 2). Average age was 57 years old. Average income was $45,000 per year, 27% lived in poverty. These women had 14 years on average of years in education; 64% of women had private health insurance, 29% of women were solely publically insured (Medicaid/Medicare) and 3% were uninsured.

**Table 2:** **Demographic characteristics**

Variable	Sample (333)	PAP (91)	No PAP (240)	T or X^2
				P value
Age	61 +/- 1.2	57+/- 2.4	62 +/- 3.3	1 p=0.323
Sum of hours	1.5+/- 0.47	4 +/- 1.6	0.4 +/- 0.2	14.7 p=000
Non-white race	53%	49%	55%	7.6 p=0.1
Years of Ed	14	15.1 +/- 0.34	13.6 +/- 0.3	7.03 p=0.008
Income < 15 k	27%	26%	28%	0.04 p=0.8
Income > 150 k	18%	14%	19%	0.9 p=0.34
Intl birth origin	41%	40%	43%	0.3 p=0.59
English	71%	79%	67%	4.4 p=0.04
Medicaid	21%	19%	22%	0.46 p=0.5
Only public insurance	6%	3%	7%	1.67 p=0.2
Medicare	4%	2%	5%	1.3 p=0.26
Any public Ins	31%	24%	34%	3.06 p=0.08
Married	49%	44%	51%	1.3 p=0.26
Utility	0.61	0.61	0.61	0.52 p=0.47
Needs	2	2	2	1.19 p=0.76
Randomized Rx	51%	56%	49%	1.4 p=0.27

Patients who attended programs were more likely to be more educated and speak English than those who did not.

**Figure 1:**
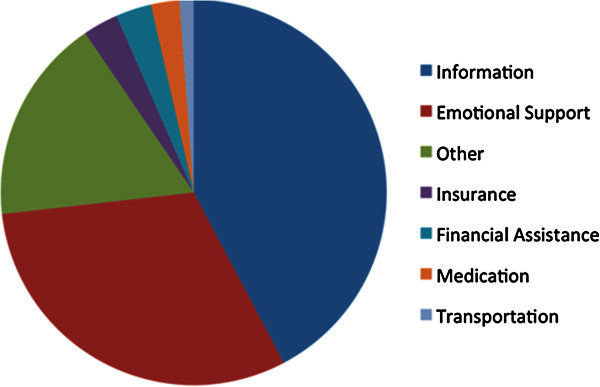
**Of the services obtained by attendees, the most common services obtained were information and social support.**

The top three accessed Patient Assistance Programs included one local Breast Health Resource Center (19%), and two national programs the American Cancer Society (15%) and Cancer Care (13%). These three programs are comprehensive offering literature, peer-support groups, lecture series, outreach breast cancer screening sessions, social work assistance/insurance application, financial assistance, transportation and medications at no cost to patients (Figure 1). Patients reported benefiting the most from information resources (71% of patients) followed by emotional support (52% of patients); 5% of patients reported getting help with insurance, 5% with financial assistance, 4% with obtaining medication, 2% with transportation. The most common source of referral to patient assistance programs was from physicians (50%), family (9%) and print material (7%).

Patients who used the programs at baseline were significantly more likely to be English speaking (p=0.04) and have 1.5 years more advanced schooling than non-utilizers (p=0.008). There was a trend for more white patients and those with private insurance to use patient assistance programs. No other differences were noted between the two groups.

### Relationship between quantity of needs, socio-demographic factors & use of patient assistance programs

Quantity of needs was significantly related to socio-demographic factors (Table 3) with greater number of needs among women who are poor (<$15,000), unmarried, minority, foreign born, Spanish speaking women with low educational attainment (≤high school), had an average of one additional need compared to married, US born, English speaking, more educated white women. 21 patients with no needs spent an average eight hours with a standard deviation of five hours in the patient assistance programs. 21 patients with one need spent an average of 27 hours with a standard deviation of 22 at patient assistance program. 15 patients with two needs spent an average of 21 hours with a standard deviation of 3 hours. 34 patients with three needs spent an average of 19 hours with a standard deviation of 18 hours. There is a trend in increased use of patient assistance programs with needs but due to the large amount of variance means that no significant difference was found in this subsample of patients.

**Table 3:** **Patient number of needs and demographic factors**

Demographic factors	m (SD) number of needs (n=333)	(F) P value
Marital Status		(9.2) 0.003
Married	1.9 (1.2)	
Not Married	1.5 (1.2)	
Educational Attainment		(46.2) 0.000
Educated <12 yrs	2.3 (1.0)	
> College	1.4 (1.2)	
Income		(60.0) 0.000
Income >150 K	0.7 (0.9)	
Income < 15 K	2.3 (1.0)	
Race		(29.7) 0.000
African American	1.9 (1.0)	
Latina	2.5 (1.1)	
White	1.1 (0.9)	
Foreign born		(83.1) 0.000
Born abroad	2.4 (1.0)	
USA born	1 (1.1)	
Language		(75.9) 0.000
Spanish speaking	2.5 (0.9)	
English speaking	1.4 (1.2)	
Public insurance		(44.6) 0.000
Medicaid enrollee	2.3 (1.0)	
No medicaid	1.3 (1.2)	

Demographic characteristics are associated with number of needs.

### Cost of existing comprehensive patient assistance programs

The average cost per hour Comprehensive Patient Assistance Programs totaled $72.90 in the 2012 USD based on the number of one hour encounters in 2007 divided by the annual expenditure (Table 1). The cost of care for each needs group varied according to the quantity of hours spent at the program. No needs group sought support costing an average of $583, where as patients with needs used $1385-1968. The cost for the abstainers was $1.32 based on mailing/print material reported cost.

## Discussion

Women with new breast cancer have a range of needs, and existing programs can meet these needs at a minimal cost. There are few published studies of existing comprehensive patient assistance programs outside the programs based on clinical trials in academic setting (Cohen et al. 2009) (Maisiak et al. 1981; Gilbar & Groisman 1991; Cella et al. 1993; Edgar et al. 1996). Of the studies that do exist, most are non-standardized surveys reporting on subjective and abstract concepts such as satisfaction and well-being rather than quantitative evaluations of cost and needs (Cella et al. 1993; Edgar et al. 1996).

Patients who attended existing comprehensive patient assistance programs were higher educated English speaking women. The majority of women reported gaining information and emotional support from these groups from access to educational materials, lectures, counseling, at the cost of $72.9 per one-hour encounter. Clinical trials have established the effectiveness of the methods such as group counseling, stress management, educational materials, physiotherapy in improving quality of life (Gordon et al. 2005; Lemieux et al. 2006; Jacobsen et al. 2002; Mandelblatt et al. 2008). Similarlarly, patient navigation programs have also been shown to prevent decline in quality of life (Giese-Davis et al. 2006). Comprehensive patient assistance programs such as Breast Health were born out of perceived need in the community (Personal Communication with Andrea Geduld). The integration of survivor volunteers in staffing is not only economically sound but lends the program credibility. An influential source of information comes from peers (Bilodeau and Degner 1996 Lorig et al. 2003; Vasas 2005; Degner et al. 1997; Mullins et al. 2004). Our data demonstrates that information and social support is what patients obtain most commonly from patient assistance programs. Volunteers communication is free of medical jargon and personally relevant from people who are culturally similar (Steinberg et al. 2006). Using lay people to disseminate information may overcome socially derived access barriers and improve quality of life in some cases by improving utilization of health care resources (Battaglia et al. 2007; David et al. 2007; Jo Anne et al. 2002). Patient assistance programs that utilize survivors can potentially become self-sustaining by attracting patients to become volunteer staff members. Despite, fiscal appeal to hospital and insurers and their potential significant impact on women’s lives, these programs remain under-utilized (Giese-Davis et al. 2006). According to our data, however, lower educated and non-English speaking women are not taking adequate advantage of these resources.

The quantity of these needs was directly related to demographic factors such as insurance status, income, and educational attainment in this economically and ethnically diverse population. The finding reinforces the validity of self-reported needs as a measure of indigence and our data demonstrate a trend that patients with needs use existing comprehensive patient assistance programs slightly more than those without need. Yet less than one third of all patients utilized these programs.

As a result, we recommend creating a matchmaking system at the time of diagnosis of early stage breast caner that would identify type and number of needs and match patients to relevant comprehensive patient assistance programs like Breast Health Resource Program. This would allow for patients with more needs to be identified and educate them about programs that can provide help.

Limitations include generalizability as NYC costs may over estimate actual cost. Patient surveyed had consented to participate in a clinical trial and may not represent the general population of women with breast cancer due to selection bias. The parent RCT educated patients to enable connection to programs, it did not prevent or strictly enforce attendance. In the end, participants did in effect, self-select whether they attended programs. This study shows who actually used these programs and what we can realistically expect women to receive from it and at what cost if these programs. The effect of patient assistance programs could not be quantified from this baseline survey data. We did not evaluate the degree of services provided and those perceived as most meaningful by patients allow us to gain a better understanding of the structure of these programs as patient resources when there is a known high degree of variability in such programs (Edgar et al. 1996).

## Conclusion

Our data suggest that existing comprehensive patient assistance programs are a low cost way to provide information, emotional support and assistance to obtain insurance, medication and transportation for women with new breast cancer. Such assistance programs present an important opportunity for vulnerable communities in our country and in countries that struggle with limited capital and infrastructure to supplement hospital-based care with community-based support.

I give my written assurance that neither the submitted material nor portions thereof have been published previously or are under consideration for publication elsewhere.

## Electronic supplementary material

Additional file 1: **Needs Assessment Tool.** (PPT 98 KB)
