# Supplementary material for: Cost of comprehensive patient assistance program in early breast cancer patients
Source: Springerplus. 2013 Apr 19;2:173. doi: 10.1186/2193-1801-2-173 (PMC3650234; doi:10.1186/2193-1801-2-173)
Supplement: Supplementary file 1 — Additional file 1: Needs Assessment Tool. (PPT 98 KB) [file 40064_2013_248_MOESM1_ESM.ppt]

## Slide 1
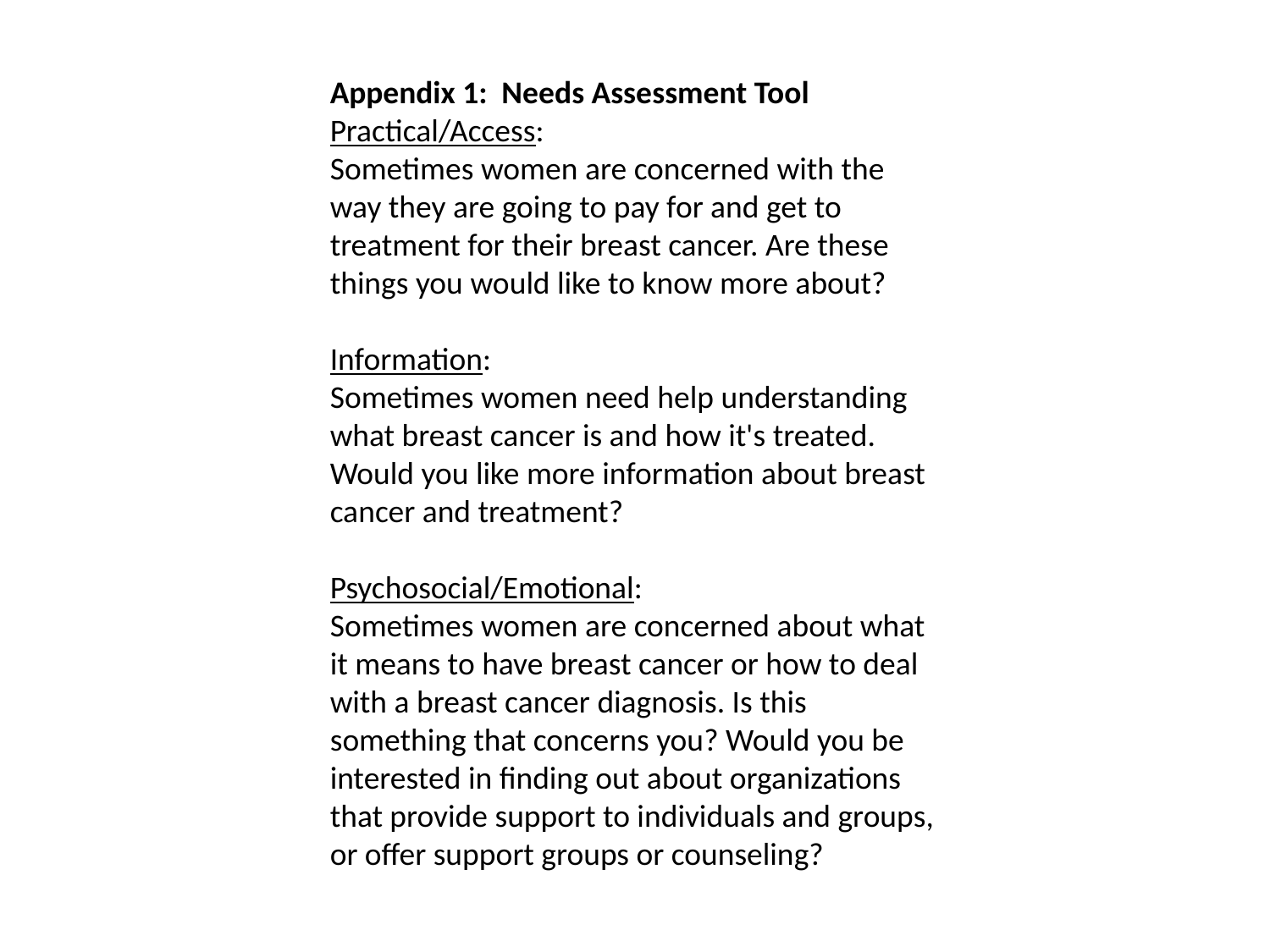

Appendix 1: Needs Assessment Tool
Practical/Access:
Sometimes women are concerned with the way they are going to pay for and get to treatment for their breast cancer. Are these things you would like to know more about?
Information:
Sometimes women need help understanding what breast cancer is and how it's treated. Would you like more information about breast cancer and treatment?
Psychosocial/Emotional:
Sometimes women are concerned about what it means to have breast cancer or how to deal with a breast cancer diagnosis. Is this something that concerns you? Would you be interested in finding out about organizations that provide support to individuals and groups, or offer support groups or counseling?
